# Artificial Intelligence-Assisted Terahertz Imaging for Rapid and Label-Free Identification of Efficient Light Formula in Laser Therapy

**DOI:** 10.3390/bios12100826

**Published:** 2022-10-05

**Authors:** Jia Shi, Zekang Guo, Hongli Chen, Zhitao Xiao, Hua Bai, Xiuyan Li, Pingjuan Niu, Jianquan Yao

**Affiliations:** 1 Tianjin Key Laboratory of Optoelectronic Detection Technology and System, School of Electronic and Information Engineering, Tiangong University, Tianjin 300387, China; 2 Key Laboratory of Opto-Electronics Information Technology (Ministry of Education), School of Precision Instruments and Opto-Electronic Engineering, Tianjin University, Tianjin 300072, China

**Keywords:** terahertz imaging, photodynamic therapy, low-level laser therapy

## Abstract

Photodynamic therapy (PDT) is considered a promising noninvasive therapeutic strategy in biomedicine, especially by utilizing low-level laser therapy (LLLT) in visible and near-infrared spectra to trigger biological responses. The major challenge of PDT in applications is the complicated and time-consuming biological methodological measurements in identification of light formulas for different diseases. Here, we demonstrate a rapid and label-free identification method based on artificial intelligence (AI)-assisted terahertz imaging for efficient light formulas in LLLT of acute lung injury (ALI). The gray histogram of terahertz images is developed as the biophysical characteristics to identify the therapeutic effect. Label-free terahertz imaging is sequentially performed using rapid super-resolution imaging reconstruction and automatic identification algorithm based on a voting classifier. The results indicate that the therapeutic effect of LLLT with different light wavelengths and irradiation times for ALI can be identified using this method with a high accuracy of 91.22% in 33 s, which is more than 400 times faster than the biological methodology and more than 200 times faster than the scanning terahertz imaging technology. It may serve as a new tool for the development and application of PDT.

## 1. Introduction

Acute lung injury (ALI) as a common lung disease with high mortality and incidence rates [1,2,3,4] is manifested by acute hypoxemic respiratory failure, increased alveolar permeability, and severe alveolar edema with normal cardiac filling pressures [5]. Due to inefficiency of traditional treatments for ALI [6], it is significant to develop a new therapeutic method for ALI to substantially reduce mortality and improve the quality of life of patients. Photodynamic therapy (PDT), as a novel treatment by provision of effective local control, is a light-activated treatment technique that harnesses a photochemical reaction on laser irradiation [7,8]. It shows potential in biomedicine as a noninvasive therapeutic strategy which demonstrated better spatial selectivity and invasiveness [9,10,11]. Low-level laser therapy (LLLT) has been extensively applied in many diseases as PDT utilizing visible and near-infrared spectra. It has succeeded in the effective treatment of inflammation [12], pain [13], and bone and tooth healing [14]. Therefore, it is significant to develop LLLT for ALI. The therapeutic effect is mainly decided by specific light formulas including light wavelength and irradiation time. However, current identification methods of efficient light formulas are complicated, time-consuming, and not very sensitive. Therefore, the development of rapid, highly sensitive, and label-free identification strategies will strongly promote the development and application of LLLT.

Common identification methods of efficient light formulas can be divided into biological methodology and radiological technology in biomedicine. The biological methodology and radiological technology are compared in biomedicine, as shown in Table 1. The therapeutic effect with different light formulas can be accurately evaluated using biological methodology but with a long time [15]. For example, biological assays according to pathological sections usually need more than 10 h. Thus, it is a major challenge to identify the therapeutic effect of LLLT in real time for efficient light formulas. The radiological diagnosis is useful in tracing the progression of the disease, which mainly includes computerized tomography (CT), magnetic resonance imaging (MRI), and positron emission tomography (PET). CT has been proven as an effective clinic tool to study the pathophysiology [16]. Due to the unavoidable radiation, CT is limited in multiple scans and in tracing the progression and regression of disease [17], and it is not sufficiently sensitive and specific for early diagnosis, quantification of cellular events, and stratification of new therapies. MRI as an early diagnosis method has the characteristics of noninvasiveness, repeatability, and dynamic observation [18]. However, its low sensitivity remains technically challenging for accurately identification of different light formulas. Most notably, hyperpolarized MRI has accuracy and high sensitivity, but it has a very short signal lifetime due to technical problems [19,20]. PET as a noninvasive radiological technology has been proposed to study a wide variety of respiratory inflammatory diseases including asthma and tuberculosis [19,21]. It may quantify molecular and cellular events in early diagnosis due to its dependence on neutrophils. However, with the progress of disease, PET will be influenced by the decreases in neutrophil activity and uptake of biomarkers. Hence, current biological methodology and radiological technology remain challenging in the identification of efficient light formulas, especially for the label-free and accurate identification of therapeutic effect.

Terahertz (THz) imaging has the properties of unique physical characteristics of a fingerprint spectrum, high diagnostic sensitivity, and safety. It has attracted great attention in biomedical applications as a promising radiological technology. It has been proposed to study a wide variety of diseases, including various cancers [22,23,24,25,26,27], skin burns [28], arthritis [29], traumatic brain injury [30], silkworm egg development stages [31], and changes in cell monolayers [32]. These findings suggest that THz imaging can quantify cellular and tissue events, as well as trace the progression and regression of disease to monitor therapeutic effect. However, THz imaging in biological diagnosis is limited by the imaging system hardware. The major bottleneck is low imaging resolution, severe blurring, and slow imaging speed. It is critical for images analysis to improve the resolution and sharpness of THz imaging, as well as reduce the imaging time. Methods of optimizing THz imaging can be divided into system optimization [33,34,35] and image reconstruction methods [36]. Blurred images are reconstructed with a high resolution and same image information using super-resolution (SR) algorithms. The method of optimizing the system is limited by the low signal-to-noise ratio of photographic imaging; thus, the SR algorithm is a better strategy. In this paper, the feasibility of the rapid THz imaging system based on the SR algorithm is proven, and THz image information is obtained for the extension of studies. With increasing image information sets in THz frequency, automatic identification technology as an efficient and rapid diagnostic tool has great potential in biological diagnoses. Recently, THz database sets were investigated for classification and recognition. Algorithms were used to distinguish cancer in THz images, including principal component analysis (PCA) [25], hierarchical clustering analysis (HCA) [37], spectroscopic integration technique [38], and support vector machine (SVM) [39]. Thus, the artificial intelligence (AI)-assisted automatic identification technology for THz imaging can significantly improve the diagnostic efficiency.

In this paper, a rapid and label-free identification method based on AI-assisted THz imaging is demonstrated to identify therapeutic effect of LLLT for ALI. The LLLT is proven as an effective therapeutic method for ALI using biological methodology. Then, a remarkable absorption peak in the range of 0.1–0.15 THz is found in the THz spectral response of ALI with potential in highly sensitive biological diagnosis and identification. A gray histogram of terahertz images at 0.14 THz is developed as the biophysical characteristics to identify the therapeutic effect. An AI-assisted identification method based on THz imaging is developed and optimized by rapid SR imaging reconstruction and automatic identification algorithm based on a voting classifier. The proposed method shows a high accuracy of 91.22% and a rapid identification time of 33 s, which is more than 400 times faster than the biological methodology and more than 200 times faster than the scanning THz imaging technology. The results indicate that the proposed method can realize the rapid and label-free identification of the efficient light formula of LLLT for ALI.

## 2. Materials and Methods

### 2.1. ALI Model in Rats

A reproducible measurement protocol was established by standardizing all experimental steps of sample preparations. Simultaneously, all animal experiments were performed in accordance with the China Animal Welfare Legislation.

**Figure 1:**
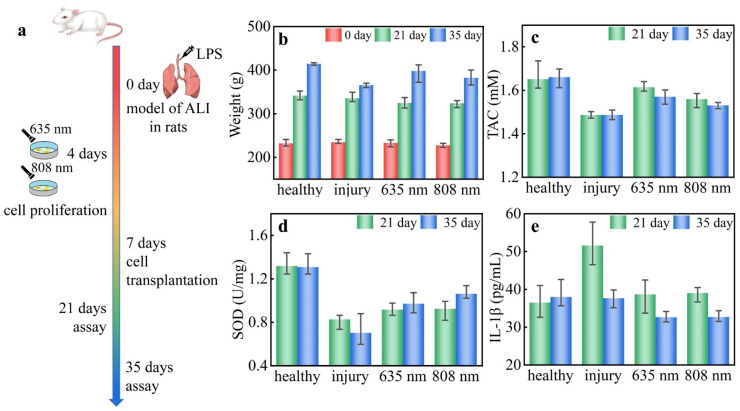
(**a**) Schematic illustration of the ALI model under LLLT. The (**b**) weight, (**c**) TAC, (**d**) SOD, and (**e**) IL-1β of the rats with ALI under LLLT.

The adult male Sprague-Dawley (SD) rats were subjected to ALI with the weight range of 220–240 g. The ALI model in rats was established using Lipopolysaccharide (LPS), as shown in Figure 1a. The rats were anesthetized by the intraperitoneal injection of 10% chloral hydrate (0.003 mL/g). The head and limbs of the rat were mounted on a frame. With the surgical site of the rat disinfected and the fur shaved, the outer skin, muscle, and fascia of the trachea were opened with an incision. With the injection of LPS (5 mg/kg) into the trachea, the rats were mounted in an upright position, and the lung tissues were fully filled to reduce the difference in modeling. As the comparison group, the same surgical procedures were also performed on healthy rats. Human umbilical cord mesenchymal stem cells (hUCMSCs) were cultured and passaged in vitro. Continuous-wave-mode laser interference was performed at an energy density of 3 J/cm^2^ and a power density of 20 mW/cm^2^ at 635 nm and 808 nm twice a day for 3 days with culture for 24 h. The cell transplantation was performed 1 week after LPS injection. The experimental data were collected after 2 and 4 weeks of treatment.

### 2.2. Super-Resolution Imaging Reconstruction Algorithms

There were four SR reconstruction algorithms used in the rapid THz imaging for the comparison of performance, namely, super-resolution generative adversarial network (SRGAN), enhanced super-resolution generative adversarial network (ESRGAN), residual channel attention network (RCAN), and enhanced deep residual network (EDSR). SRGAN introduces the generative adversarial network (GAN) to the SR domain, providing a powerful framework for SR reconstruction [40]. ESRGAN enhances the visual quality by improving each of the three key components of the SRGAN, i.e., network architecture, adversarial loss, and perceptual loss [41]. The representation capability of convolutional neural networks is improved by the RCAN, thereby improving the image quality of reconstruction [42]. With the development of deep convolutional neural networks, EDSR as a residual learning technology improves the performance of image reconstruction [43].

The performance of the SR imaging reconstruction algorithms can be evaluated using the mean square error (MSE), peak signal-to-noise ratio (PSNR), and structural similarity (SSIM). The MSE and PSNR were calculated as follows [44]:

(1) $$\mathrm{MSE}=\frac{1}{M}\sum_{i=0}^{m-1}\sum_{j=0}^{n-1}{\left[X\left(i,j\right)-Y\left(i,j\right)\right]}^{2},$$

(2) $$\mathrm{PSNR}=10•\mathrm{lg}(\frac{255^{2}}{\mathrm{MSE}}),$$

where M is the size of image X and image Y. MSE is used to evaluate the disparity of two images, and PSNR is used to evaluate the imaging quality.

The SSIM can be calculated as follows [45]:

(3) $$\mathrm{SSIM}\left(x,y\right)=\frac{\left(2\mu_{x}\mu_{y}+c_{1}\right)\left(2\sigma_{xy}+c_{2}\right)}{\left(\mu_{x}^{2}+\mu_{y}^{2}+c_{1}\right)\left(\sigma_{x}^{2}+\sigma_{y}^{2}+c_{2}\right)},$$

where c_1_ and c_2_ are constants, μ_x_ and μ_y_ are the mean value of x and y, respectively, σ_x_ and σ_y_ are the standard deviation of x and y, respectively, and σ_xy_ is the covariance of x and y. SSIM is used to evaluate the similarity of two images, which includes luminance, contrast, and structure.

### 2.3. Automatic Identification Algorithms

In the identification method, PCA as the feature extraction algorithm is applied to the gray histograms of THz images. Then, there are three algorithms used in the automatic identification based on machine learning, namely, *k*-nearest neighbor (*k*NN), random forest (RF), and SVM. Then, a voting classifier is developed using these classifiers. In the feature extraction of THz images, PCA is applied to map the *n*-dimensional features to the *k*-dimension features in new orthogonal features which is known as principal component [46]. In the *k*NN classification, the object is classified as a class if major of the *k*-nearest neighbors in the feature space belong to that class [47,48]. *k*NN is suitable for automatic classification of classes with relatively large sample sizes. RF classification is a classifier that contains multiple decision trees, and its result belongs to the major class of all results of individual decision trees [49,50]. It is appropriate for large samples and unmarked pattern recognition. SVM is a class of generalized linear binary classifiers that perform classification of data in a supervised learning manner [51,52]. It maps feature vectors to a feature space and constructs maximally spaced hyperplanes to make separation in the space easier. It is suitable for small sample, nonlinear, and high-dimensional pattern recognition.

Furthermore, the receiver operating characteristic curve (ROC) and area under curve (AUC) are used to evaluate the generalization ability of the identification algorithms. The ROC curve is correlated with the false positive rate (FPR) and true positive rate (TPR). In order to demonstrate the performance of classifier, the identification results are evaluated as a function of sensitivity, specificity, and F1-score. The TPR, FPR, sensitivity, specificity, and F1-score can be calculated as follows [53,54]:

(4) $$\mathrm{TPR}=\mathrm{Sensitivity}=\mathrm{Recall}=\frac{\mathrm{TP}}{\left(\mathrm{TP}+\mathrm{FN}\right)},$$

(5) $$\mathrm{FPR}=\frac{\mathrm{FP}}{\left(\mathrm{FP}+\mathrm{TN}\right)},$$

(6) $$\mathrm{Specificity}=\frac{\mathrm{TN}}{\left(\mathrm{TN}+\mathrm{FP}\right)},$$

(7) $$\mathrm{Precision}=\frac{\mathrm{TP}}{\left(\mathrm{TP}+\mathrm{FP}\right)},$$

(8) F1-score=2*Precision*Recall(Precision+Recall),

where TP, TN, FP, and FN are the true positive, false positive, false negative, and true negative, respectively. The AUC score is the result of the integration of the ROC curve.

## 3. Results and Discussion

### 3.1. Therapeutic Effect of LLLT for ALI

In this section, the therapeutic effect of LLLT for ALI is identified according to the biological methodological parameters, including levels of weight, total antioxidant capacity (TAC), superoxide dismutase (SOD), and interleukin-1β (IL-1β).

The weight changes of the rats are shown in Figure 1b. The weight was 232 ± 6 g before LPS modeling in each group. After 21 days of modeling, there was no obvious difference in increased weight in different groups, which was 331 ± 10 g. After 35 days of modeling, the smallest increase in weight of 30 ± 5 g was observed in the injury group, and the largest increase in weight of 73 ± 3 g was observed in the healthy group. It is shown that the weight decline was caused by ALI for the rats, but it recovered to the healthy group after LLLT especially in the 635 nm treated group. The TAC of the rats is shown in Figure 1c, which was obviously lower in the injury group. The LLLT groups demonstrated a decreasing trend with time. After 21 days of modeling, the TAC of rats in the LLLT groups recovered to the level of the healthy group, i.e., 1.60 ± 0.05 mM. After 35 days of modeling, a decreased TAC of 1.55 ± 0.04 mM was observed in the LLLT groups, but this was higher than the injury group, i.e., 1.48 ± 0.02 mM. The TAC indicates that the overall antioxidant capacity of the rats was improved by LLLT, because the LLLT can balance the oxidative stress and establish a conducive microenvironment of recovery [15]. The SOD of the rats is shown in Figure 1d. The total SOD obviously declined after LPS modeling. After 21 days of modeling, the SOD was 0.92 ± 0.07 U/mg in the LLLT groups. After 35 days of modeling, the SOD was 0.98 ± 0.08 U/mg in the LLLT groups but only 0.70 ± 0.17 U/mg in the injury group. The result of SOD indicates that the LLLT can increase the activity of antioxidant enzymes in tissues and further balance the oxidative stress state. The IL-1β level of rats is shown in Figure 1e. After 21 days of modeling, there was an obvious increase in IL-1β from 36.5 to 51.6 pg/mL in the injury group, but it recovered to 38.9 ± 4.8 pg/mL in the LLLT groups. After 35 days of modeling, the IL-1β of rats of the LLLT groups demonstrated a decreasing trend, i.e., 32.6 ± 1.5 pg/mL. The IL-1β result indicates that the LLLT was effective in slowing down the burst of proinflammatory factors during treatment. Furthermore, the secretion of proinflammatory factors was reduced in the long term, and the secondary injury due to inflammatory response was slowed down by LLLT [15].

In this section, the biological methodology proved that the LLLT is an effective therapeutic method for ALI. It took more than 4 h for the identification of efficient light formulas with different light wavelengths and irradiation times using biological methodological parameters, including weight, TAC, SOD, and IL-1β.

### 3.2. Label-Free THz Imaging of ALI under LLLT

THz spectral diagnosis has been proven to be a powerful tool for many diseases with unique physical characteristics in terms of the fingerprint spectrum, high sensitivity, and safety. Here, the diagnostic and identification capacity of the THz spectra for ALI was demonstrated using a reflective terahertz-time domain spectroscopy (THz-TDS) system (Advantest, TAS7500TS).

**Figure 2:**
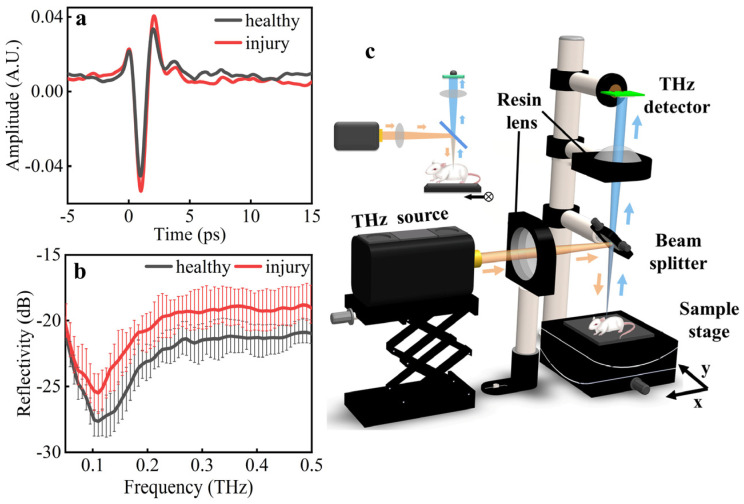
Experimental measurement of THz (**a**) time-domain waveforms and (**b**) reflective spectra of ALI samples. (**c**) Optical design of reflective THz imaging system for ALI.

Two THz pulses were registered by the THz-TDS system. One passed through the healthy lung tissue as the reference and the other passed through the injury lung tissue for diagnosis. As shown in Figure 2a, the average amplitude of the healthy tissues was smaller than that of the injury tissues. The frequency-domain spectra were obtained using a fast Fourier transformation of the time-domain signals, as shown in Figure 2b. Within the frequency range of 0.05–0.5 THz, the reflectivity of the healthy tissues was distinctly lower than that of the injury tissues. Furthermore, the spectral measurement of the THz-TDS demonstrated a remarkable absorption peak in the range of 0.1–0.15 THz with the potential in highly sensitive diagnosis and identification of ALI.

With consideration of the highly sensitive THz spectral response of ALI, a vertical reflective THz imaging system was designed for label-free imaging at 0.14 THz. As shown in Figure 2c, the THz radiation source (Terasense, IMPATT diodes, 20 mW) was used with an ultrafast THz detector (Terasense, 50 GHz–0.7 THz) to enable the highly sensitive imaging of ALI in the system. The front view of the imaging system is shown in the subgraph. The emitted THz waves were delivered by the resin lens (*f* = 100 mm) to the beam splitter and vertically focused on the animal (yellow arrow). The reflected THz waves were delivered from the beam splitter to the resin lens (*f* = 100 mm) and focused on the THz detector (blue arrow). The animal was fixed on a computer-controlled linear motor stages for the two-dimensional raster scan imaging. The resin lenses were made using 3D printing, and the size of the focal spot was 3 mm, measured using the knife-edge method. The imaging speed was about 10 pixels/second.

**Figure 3:**
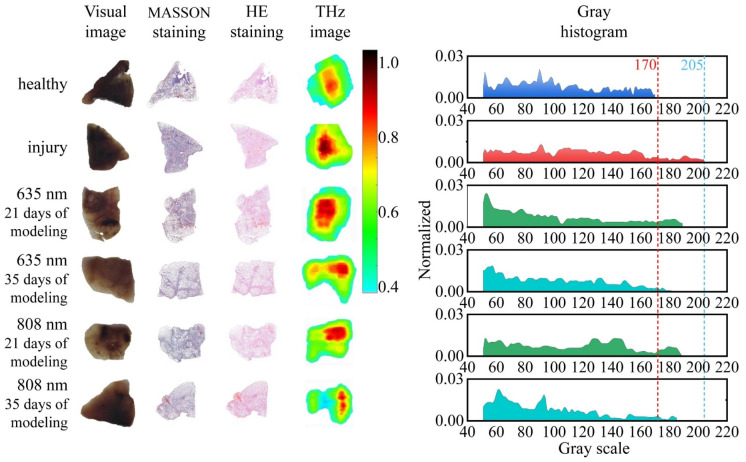
Images of the ALI samples at different stages of LLLT including visual images, MASSON staining images, HE staining images, THz images, and corresponding gray histograms of THz images.

The highly sensitive THz images and their gray histogram characteristics for ALI under LLLT were further analyzed in comparison with the visual images and histopathologic staining images. Six paraffin-embedded ALI samples were assayed, and all the experiments were performed in temperature- and humidity-equilibrated laboratories. The image table of the six samples in Figure 3 shows the visual images, MASSON staining images, HE staining images, THz images, and corresponding gray histograms of THz images, respectively.

The visual images show the paraffin-embedded ALI tissues under LLLT with different light wavelength and irradiation time. The injury degree and therapeutic effect of LLLT for ALI could not be identified through the visual images. The histopathologic analyses of MASSON and HE staining images were further established to visually analyze the cell density. The results of histopathologic analyses indicated that injury tissues had a higher cell density compared with the healthy tissues. The LLLT could improve the microenvironment of cell and reduce the cell density, especially at the wavelength of 635 nm. The increase in cell density in ALI tissues mainly resulted from multiple biological events including the alveolar interstitial inflammatory cell infiltration and bleeding [5].

The cell density has been proven as a high-sensitive diagnostic indicator of the injury area in THz biomedical imaging in several studies [55]. In this paper, the THz images of ALI were assembled with the reflectivity of the acquired THz signal and compared with the cell density. An imaging area of 3 × 3 cm was obtained with scanning steps of 100 μm. In order to analyze the healthy and injury tissue quantitatively, the reflectivity was used in the THz images normalized by the intensity of THz signal reflected from the paraffin. The THz images indicated that the intensity of reflective THz signal from the injury area was distinctly greater than that from the healthy area, corresponding to the cell density in the histopathologic staining images.

Then, the gray histograms of the THz images were developed to reflect the biophysical characteristics in the analysis of injury degree. The gray histograms demonstrated that the distribution range of injury samples (about 40–205) was distinctly wider than that of healthy samples (about 40–170), which can be explained by the gray distribution of injury tissues in THz images being mostly focused in the range from 170 to 205 due to its higher cell density. Further comparing the LLLT for ALI with different light wavelengths and irradiation times, the gray histograms indicated that the gray distribution of injury samples presented a remarkable trend of recovery to the healthy samples. Moreover, the longer modeling time brought about a better recovery effect. When the modeling time was within 21 days, there was a similar recovery effect using the 635 nm and 808 nm laser. With the modeling time reaching 35 days, the recovery effect using the 635 nm laser was better than that using the 808 nm laser and almost recovered to the healthy samples.

In this section, THz imaging was proven as a highly sensitive and label-free method in the identification of efficient light formulas for ALI. The scanning THz imaging technology needed about 2 h for each ALI sample in the imaging, which was about half time of the biological methodological measurements. The gray histogram of THz images demonstrated its potential as a universal biophysical characteristic in biomedical identification.

### 3.3. Rapid Identification of Light Formulas Using AI-Assisted THz Imaging

The scanning THz imaging technology had a high imaging resolution and only took half the time of the biological methodological measurements in identification of light formulas; however, it is still difficult to meet the demand of identification in real time. The THz camera imaging technology has a high imaging speed, but its low imaging resolution usually limits its biomedical applications. By increasing the scanning step of the scanning THz imaging system, the imaging time can be significantly reduced but with a decline in imaging resolution. Here, a rapid THz imaging technology-based SR imaging reconstruction was developed to further reduce the imaging time without compromising imaging resolution.

The method involves first obtaining low-resolution THz images with a large scanning step in a short time, and then reconstructing high-resolution THz images from the low-resolution images using rapid SR imaging reconstruction algorithms. In order to analyze its performance, some reconstruction methods are demonstrated for the THz images with different scanning steps.

**Figure 4:**
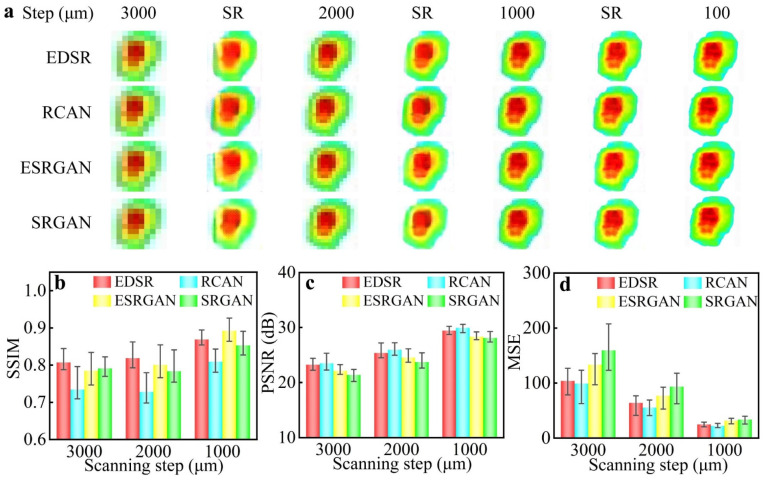
(**a**) THz images with different scanning steps and SR reconstructed images using different algorithms. (**b**–**d**) SSIM, PSNR, and MSE of the SR reconstructed images.

The original scanning images and corresponding SR reconstructed images obtained using different algorithms are shown in Figure 4a. The scanning time of the THz image was decreased from 2 h to 10 s as the scanning step increased from 100 μm to 3000 μm. With a scanning step of 3000 μm, the phenomenon of structural destruction and color chroma degradation was observed in the reconstructed images with lattices and blurring artefacts. In contrast, the image quality was improved distinctly in the reconstructed images with other scanning steps. As the scanning step decreased from 3000 μm to 1000 μm, the scaling factor of reconstruction decreased, and the image quality improved with the scanning time increasing from 10 s to about 90 s. The result shows that these SR imaging reconstruction algorithms succeeded in restoring the structure and color chroma of THz images when the scanning step was less than 2000 μm. In order to further analyze the reconstruction algorithms quantitatively, the indicators of SSIM, PSNR, and MSE were calculated, as shown in Figure 4b–d. It is worth mentioning that these indicators can provide a significant reference for algorithm selection. With the scaling factor of reconstruction decreasing, the indicators of the reconstruction algorithms can be improved. The SSIM <0.80 suggests that the structure of RCAN did not reconstruct the original structure. The EDSR outperformed other algorithms with a PSNR >25 and SSIM >0.82.

In consideration of imaging time and reconstructed quality, the EDSR algorithm with a scanning step of 2000 μm was used in the rapid THz imaging system, which reduced the scanning time to 25 s. The reconstruction time of the algorithm was less than 5 s. Using this rapid reconstruction method, each THz image could be obtained in 30 s without compromising imaging resolution, which is more than 200 times faster than the scanning THz imaging system.

Although the THz images can be acquired in 30 s using the proposed rapid THz imaging system, the biological identification of THz radiography is still limited by highly professional experience. With assisted by automatic identification method based on machine learning, the requirement of identification for professional experience is reduced significantly. Currently, the recognition rate of the common algorithms struggles to satisfy requirements. Thus, it is necessary to develop an appropriate learning algorithm for the biological THz images.

**Figure 5:**
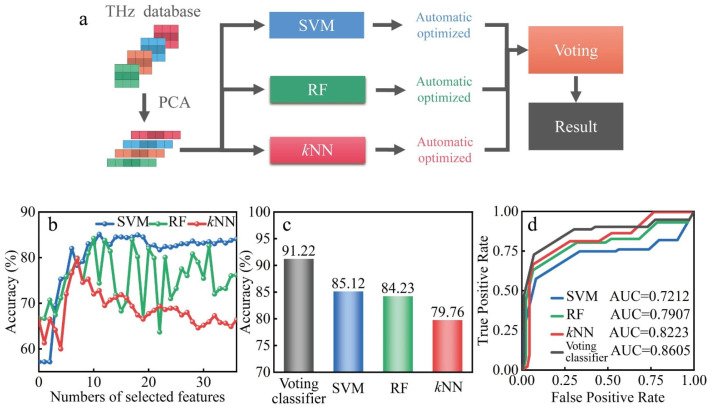
(**a**) Flowchart of the proposed voting classifier for the biological THz images. (**b**) The classification accuracy of *k*NN, SVM, and RF with the different numbers of features. (**c**) The highest classification accuracy of different classifiers. (**d**) The ROC curve and AUC scores of different classifiers when they reach the highest accuracy.

A voting classifier based on multiple classifiers is proposed with the flowchart shown in Figure 5a. The THz dataset constituted the 728 THz images obtained from the animal ALI model under LLLT with different light formulas, which included 104 images from the healthy group, 208 images from the injury group, 208 images from the 635 nm-treated group, and 208 images from the 808 nm-treated group. The gray histograms were extracted from the THz images as the feature parameters with the dimensionality reduction by PCA, and then identified using the classifiers of *k*NN, SVM and RF, respectively. Lastly, the final identification result was obtained according to the voting result of these three classifiers. In order to improve the performance of the voting classifier, the identification indicators of these three classifiers were optimized by automatic ergodic combination [30]. The identification accuracy obtained from individual classifiers with different numbers of selected features is shown in Figure 5b. The highest accuracy of individual classifiers was 85.12%, 84.23%, and 79.76%, respectively. Using the voting classifier, the identification accuracy was improved significantly to 91.22%, as shown in Figure 5c. As shown in Figure 5d, when different classifiers reached the highest accuracy, the ROC curve and AUC scores were analyzed to further demonstrate the performance of generalization ability. It is worth mentioning that the voting classifier showed a better generalization ability with the AUC score reaching 0.8605. Then, the sensitivity, specificity, and F1-score were calculated to demonstrate the performance of voting classifier, as shown in Table 2. Compared with other individual classifiers, the result indicates that the voting classifier had outstanding ability in terms of sensitivity, specificity, accuracy, and generalization ability. The identification time of the voting classifier was less than 3 s.

In this section, the AI-assisted THz imaging was sequentially performed using rapid SR imaging reconstruction and an automatic identification algorithm based on a voting classifier. It took 30 s for the imaging of each ALI sample and 3 s for the identification algorithm of therapeutic effect. Therefore, the identification time of the proposed method was 33 s for each biological sample, which is more than 400 times faster than the biological methodological measurements and more than 200 times faster than the scanning THz imaging system.

## 4. Conclusions

In summary, a rapid and label-free identification method based on THz imaging was performed to identify the therapeutic effect of ALI under LLLT with different light wavelengths and irradiation times. The LLLT was proven as an effective therapeutic method for ALI using biological methodology. Then, a remarkable absorption peak in the range of 0.1–0.15 THz was found in the THz spectral response of ALI with potential in highly sensitive biological diagnosis and identification. The gray histogram of THz images at 0.14 THz was developed to reflect the biophysical characteristics and identify the therapeutic effect. An AI-assisted identification method based on THz imaging was developed and optimized using rapid SR imaging reconstruction and an automatic identification algorithm based on a voting classifier. The proposed method showed a high accuracy of 91.22% and a rapid identification time of 33 s, which is more than 400 times faster than the biological methodology and more than 200 times faster than the scanning THz imaging technology. In the future, the compressed sensing and super-resolution algorithm can be combined to optimize the AI-assisted THz imaging. The AI-assisted THz imaging in this paper was only used for the identification of paraffin-embedded samples of biological tissues. Further studies can be performed using in vivo imaging of fresh biological samples. AI-assisted THz imaging represents a powerful tool for the development and application of PDT, and it can be extended to other biological applications.

## Figures and Tables

**Table 1:** The pros and cons of biological methodology and radiological technology in biomedicine.

Methods	Biological Methodology [15]	CT [16,17]	Hyperpolarized MRI [18,19,20]	PET [19,21]
Pros	Accurate	Clinic tool	Accuracy and high sensitivity	Accurate and high-sensitive
Cons	Complex and time-consuming	Unavoidable radiation and low sensitivity	Short signal lifetime	Influence of the progresses of disease

**Table 2:** The identification metrics of AI-assisted THz imaging.

	Voting Classifier	SVM	RF	*k*NN
Sensitivity	0.8872	0.8125	0.7483	0.8038
Specificity	0.9403	0.8765	0.8910	0.8959
F1-score	0.9060	0.7919	0.7912	0.8239

## Data Availability

Not applicable.
